# Supplementary material for: A qPCR Assay for the Fast Detection and Quantification of Colletotrichum lupini
Source: Plants (Basel). 2021 Jul 28;10(8):1548. doi: 10.3390/plants10081548 (PMC8401954; doi:10.3390/plants10081548)
Supplement: Supplementary file 1 [file plants-10-01548-s001.zip › plants-1276674-supplementary.pdf]

plants

MDPI

Article

A qPCR Assay for the Fast Detection and Quantification of *Colletotrichum lupini*

Tim Kamber [1,†], Nachelli Malpica-López [2,†], Monika M. Messmer [1], Thomas Oberhänsli [1], Christine Arncken [1], Joris A. Alkemade [1] and Pierre Hohmann [1,*]

Supplementary Materials

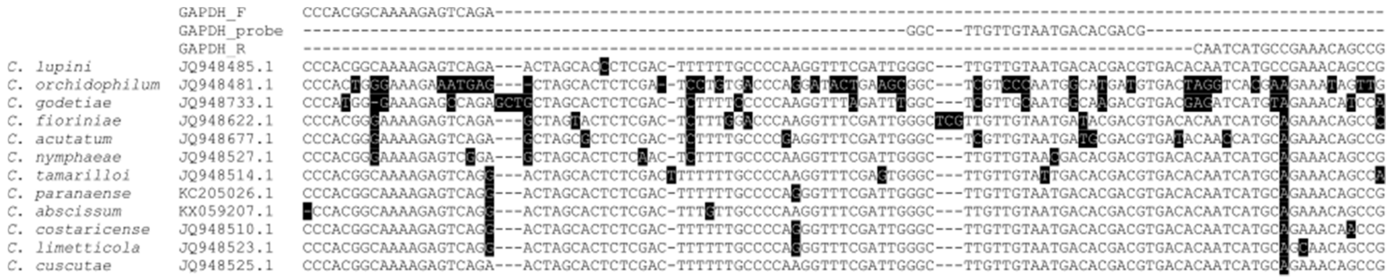

Figure S1. Multiple alignment of the target region of the GAPDH gene from different *Colletotrichum* species.

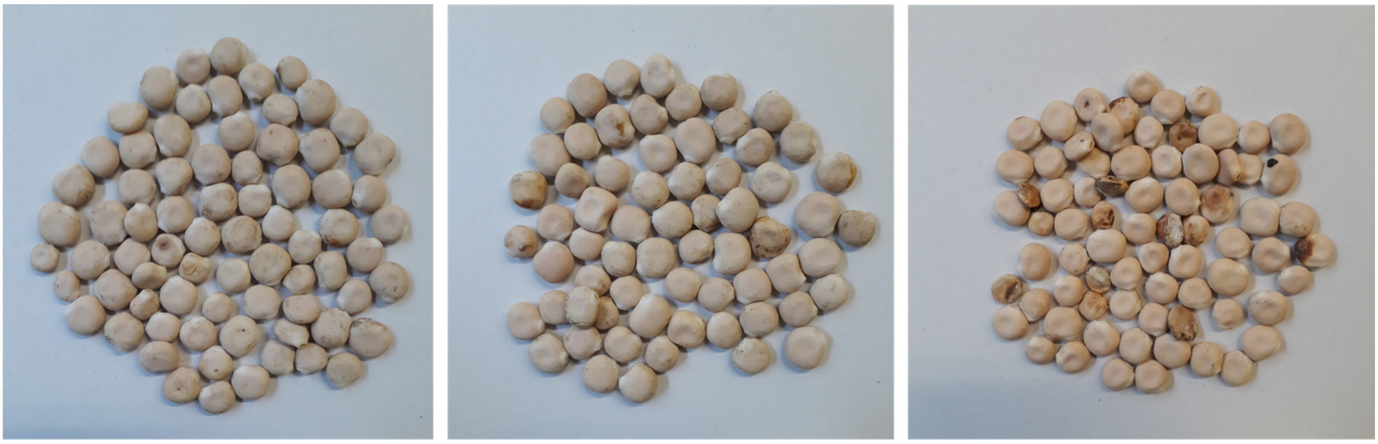

W39 W63 L9

Figure S2. Seed batches used in this study.
